# Improving quality of life after breast cancer: a comparison of two microsurgical treatment options for breast cancer-related lymphedema (BCRL)

**DOI:** 10.1007/s10238-024-01344-w

**Published:** 2024-04-23

**Authors:** Katrin Seidenstuecker, Sonia Fertsch, Alina A. Ghazaleh, Adriano Fabi, Julia Stoffel, Julia Bukowiecki, Andreas Wolter, Soheila Aghlmandi, Anshoo Nadella, Florian S. Halbeisen, Christoph Andree, Martin D. Haug, Dirk J. Schaefer, Tristan M. Handschin, Elisabeth A. Kappos

**Affiliations:** 1 Department of Plastic, Reconstructive and Aesthetic Surgery, Sana Hospital Düsseldorf, Düsseldorf, Germany; 2 Breast Center, University Hospital Düsseldorf, Düsseldorf, Germany; 3 Faculty of Health, University Witten-Herdecke, Witten, Germany; 4 Department of Plastic, Reconstructive, Aesthetic and Hand Surgery, University Hospital of Basel, Spitalstrasse 21, 4031 Basel, Switzerland; 5 Faculty of Medicine, University of Zurich, Zurich, Switzerland; 6 Faculty of Medicine, University of Basel, Basel, Switzerland; 7 Surgical Outcome Research Center, Department of Clinical Research, University Hospital Basel and University of Basel, Basel, Switzerland; 8 Breast Center, University Hospital of Basel, Basel, Switzerland

**Keywords:** Breast cancer-related lymphedema, Breast cancer, Lymphatic surgery, VLNT, LVA, Supermicrosurgery

## Abstract

**Purpose:**

Vascularized lymph node transfer (VLNT) entails the autologous relocation of lymph nodes to a lymphedematous region of the body, whereas lymphaticovenous anastomosis (LVA) creates a direct bypass between the lymphatic and venous system. Both techniques are meant to lastingly bolster the local lymphatic drainage capacity. This study compared safety and effectiveness of VLNT and LVA in patients with chronic breast cancer related lymphedema (BCRL).

**Methods:**

A retrospective cohort study was conducted using data from our encrypted database composed of patients with chronic BCRL who were treated with either VLNT or LVA and had a minimum follow-up of two years. Patient-specific variables analyzed included pre- and postoperative arm circumferences, lymphedema stages and postoperative complications.

**Results:**

A total of 105 patients met the inclusion criteria, of which 96 patients demonstrated a complete follow-up period of two years. The VLNT group displayed larger preoperative circumferential measurements, evident in both in the isolated examination of the affected arm, as well as when adjusted for the contralateral unaffected arm. Significant reduction in arm volume was achieved by both groups. However, VLNT demonstrated superior relative reduction rates than LVA, neutralizing any significant arm size disparities after 24 months. Surgery duration was slightly longer for VLNT than LVA. Postoperative complications, predominantly minor, were exclusively observed in the VLNT group.

**Conclusions:**

Both VLNT and LVA offer significant improvement for patients suffering from chronic BCRL. VLNT shows an even greater potential for improvement in more severe cases of BCRL, but involves a higher risk for (mostly minor) complications.

## Introduction

Chronic breast cancer-related lymphedema (BCRL) affects more than one in five breast cancer survivors [1], often resulting in physical and psychological repercussions, such as limb pain and numbness, diminished grip strength, body dysmorphia and depression [2–10]. While conservative treatment methods, namely complex physical decongestion therapy (CDT), provide symptomatic relief, their clinical effectiveness remains limited [11, 12]. Microsurgical interventions, on the other hand, offer promising therapeutic effects by addressing the underlying pathomechanism of chronic BCRL [13]. Vascularized lymph node transfer (VLNT) entails the autologous microvascular relocation of lymph nodes from an unaffected area of the body, e.g. the groin or intra-abdominal sites, to a lymphedematous site. They are then connected to recipient vessels via vascular anastomosis [14–16]. Conversely, lymphaticovenous anastomosis (LVA) establishes a bypass by anastomosing lymphatic vessels directly to neighbouring subdermal venules, thereby allowing the lymphatic flow to channel into the venous system [17–19]. Several studies have validated the effectiveness of both techniques in bolstering the local lymphatic drainage capacity [15, 20–24]. Therefore, the main objectives of this study were to compare VLNT with LVA in regards to patient outcomes and the incidence of postoperative complications.

## Methods

### Surgical procedure: vascularized lymph node transfer

VLNT surgery involves the autologous transplantation of intact lymph nodes to a lymphedematous region of the body [25]. In this study, lymph nodes harvested from the groin, mesentery or omentum were transplanted to the axillary region, following scar removal at the recipient site. Once transferred, the flap underwent microsurgical anastomosis using 9–0 or 10–0 Ethilon stitches. The flap's viability was confirmed by Doppler signals and observation of punctual bleeding of the corium, after which it was properly positioned and secured with 3–0 Vicryl sutures.

### Surgical procedure: lymphaticovenous anastomosis

In LVA, approximately 2 cm skin incisions are made on the affected arm to identify functional lymphatic vessels, which are then anastomosed to adjacent efferent veins in the subcutaneous tissue [26]. During this procedure, a high-magnification microscope and specialized supermicrosurgical instruments and sutures were used, given that lymphatic vessels can have diameters of less than 0.8 mm [27]. In the majority of our cases, side-to-end anastomosis was performed with 11–0 Ethilon stitches in supermicrosurgery technique [28].

### Study design

Characteristics of all patients treated for BCRL at a tertiary referral center were entered into an encrypted database. The following inclusion criteria were then applied: patients treated for chronic BCRL with either VLNT or LVA between Jan 1st, 2015, and Dec 31st, 2022, with a minimum follow-up of two years and a written informed consent.

The international society of lymphology’s definition of chronic lymphedema (inter-limb difference of over 10% in volume or excess volume between the lymphedematous and unaffected arm present for more than three months) was used to define chronicity in the prospectively maintained database including all lymphedema patients [29]. However, this study included patients with BCRL only.

To objectively assess the severity of chronic BCRL, circumferential measurements served as clinical surrogates. For each patient, circumferences of both the lymphedematous and the unaffected arm were measured at distinct reference points. These reference points were defined as the level of the thumb saddle joint, the wrist, and additional points at 10 cm, 20 cm, 30 cm, 40 cm and 50 cm proximal to the wrist [25, 30]. Postoperative measurements after 24 months were compared to preoperative values. Additionally, lymphedema stages and postoperative complications classified according to Clavien-Dindo were recorded [29, 31, 32].

### Relative reduction rates

To accurately assess potency accounting for changes in body size over time, relative reduction rates (RRR) of the circumferences were calculated compared to the preoperative measurements [33]. The following formula was used to calculate relative reduction rates:

$$RRR = \left( {1 - \frac{{ \frac{{\emptyset \;{\text{Circumference at }}\;24{\text{ months }}\left( {\text{affected arm}} \right)}}{{\emptyset \;{\text{Circumference preoperative }}\left( {\text{affected arm}} \right)}}{ }}}{{ \frac{{\emptyset \;{\text{Circumference at}}\;{ }24{\text{ months }}\left( {\text{unaffected arm}} \right)}}{{\emptyset \;{\text{Circumference preoperative }}\left( {\text{unaffected arm}} \right)}} }}} \right)*100$$.

### Statistical analysis

All statistical analyses were performed using R software (version 4.2.2) with *p*-values ≤ 0.05 indicating statistical significance. Patients’ characteristics were analyzed as mean and standard deviation (SD) and 95% confidence intervals. Independent two-sample t-tests were performed to compare arm circumferences of the affected and unaffected arms at distinct reference points, along with their differences, between the VLNT and LVA patient groups.

## Results

In this study comprising 105 patients, 100 (95.2%) patients were female and 5 (4.8%) were male. Among these, 58 (55.2%) patients underwent VLNT surgery, while 47 (44.8%) patients had LVA surgery. VLNT was performed using inguinal lymph nodes in 56 (96.6%) patients and abdominal lymph nodes in 2 (3.4%) patients, of which one was mesenteric and the other omental. LVA surgery, on average, incorporated 2.4 ± 0.7 bypasses, predominantly (97.8%) of the side-to-end type.

Both groups showed similar mean ages of 54.0 ± 9.8 years and 53.7 ± 11.3 years, respectively, and comparable BMIs, with 27.1 ± 3.9 in the VLNT and 27.0 ± 5.3 in the LVA group. In the VLNT group, 54 (93.2%) patients had stage II and two (3.4%) patients had stage III chronic BCRL. The preoperative stage of lymphedema was not documented for the remaining two (3.4%) patients. Similarly, 44 (93.6%) patients treated with LVA had stage II chronic BCRL. The preoperative stage was not documented for the remaining three (6.4%) patients. The minimum follow-up period of two years was achieved by 96 (91.4%) patients. The remaining nine (8.6%) patients were excluded from subsequent circumferential calculations.

**Fig. 1:**
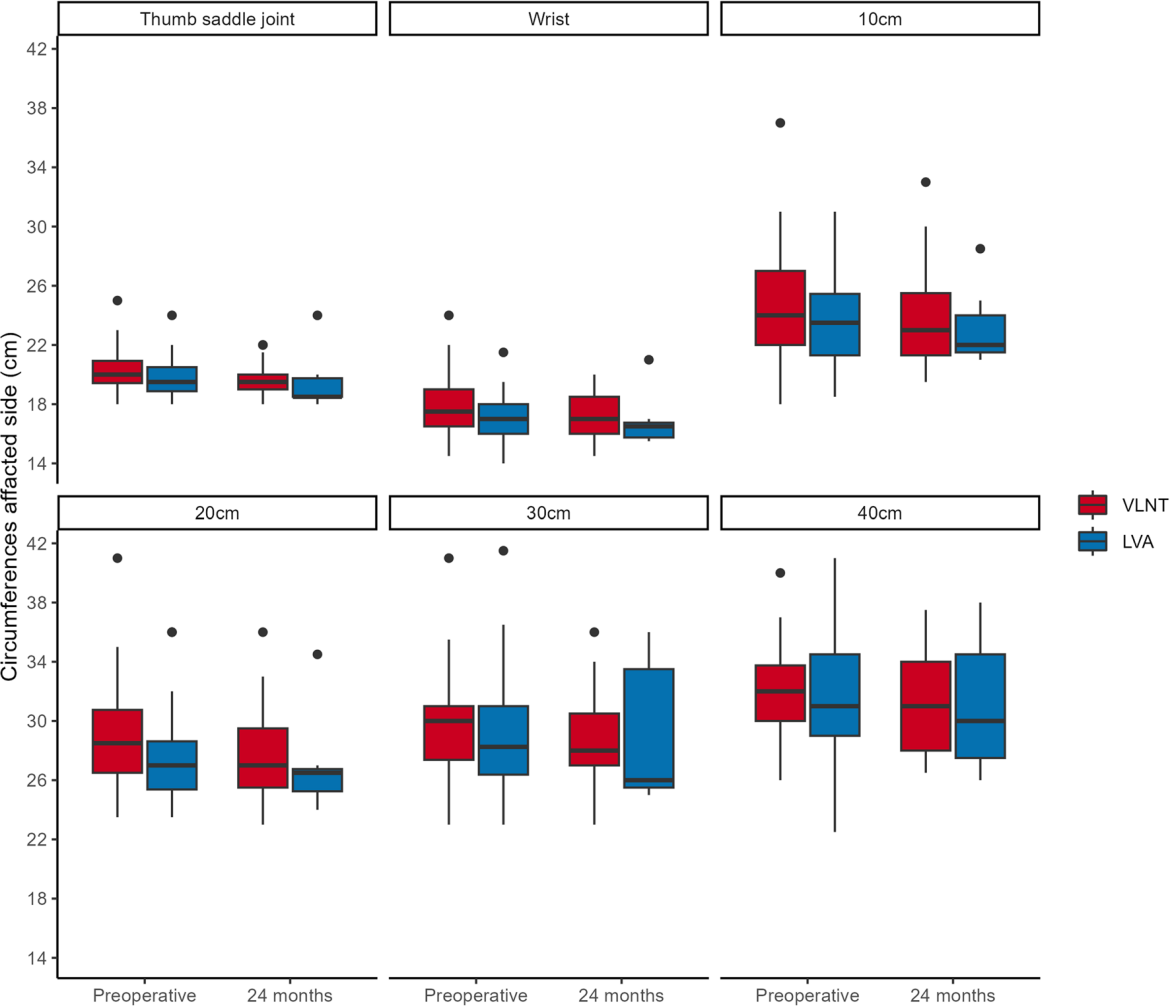
Comparison of pre- and postoperative circumferences of the affected arm

**Fig. 2:**
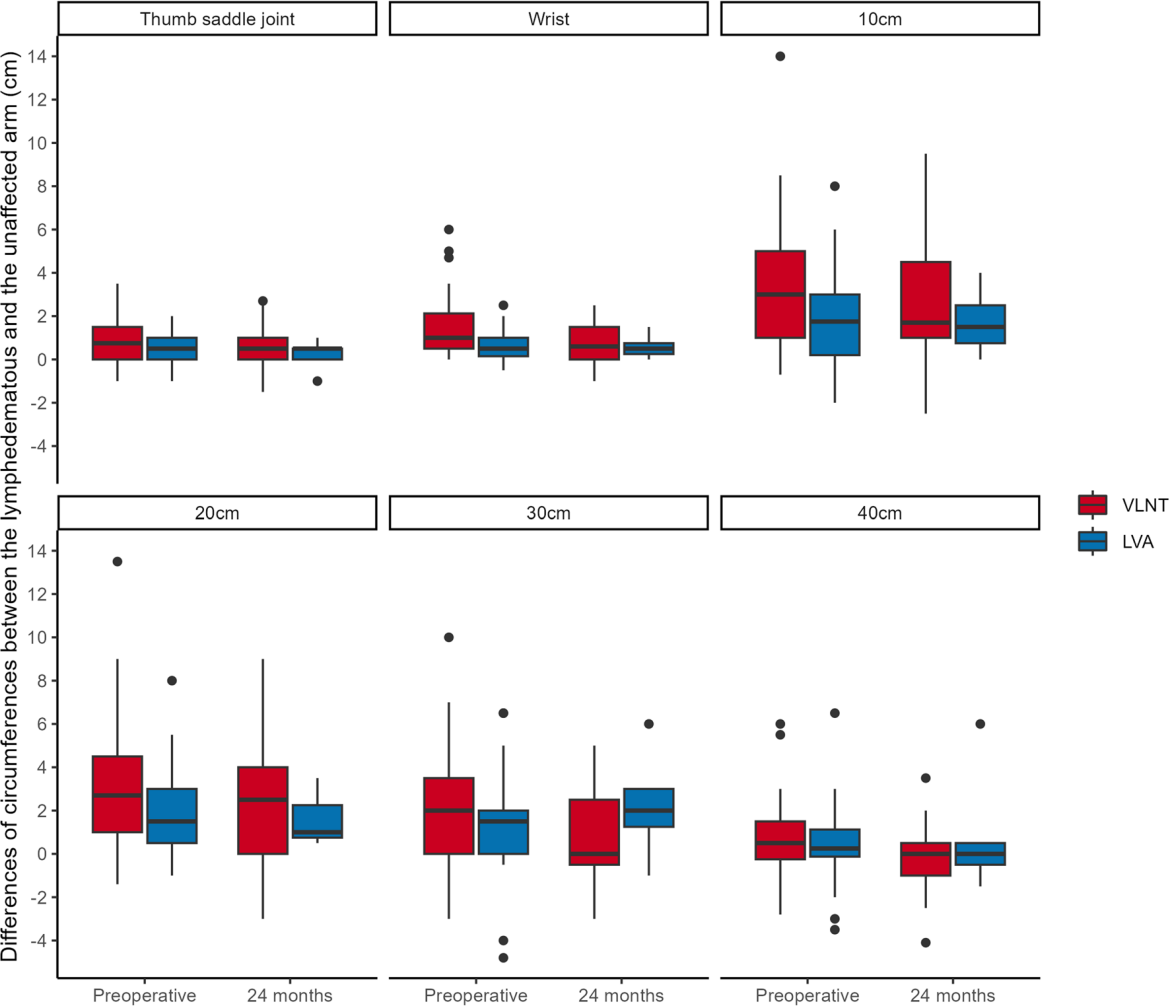
Comparison of pre- and postoperative differences of circumferences between the lymphedematous and the unaffected arm

Preoperative circumferential measurements of the unaffected arm demonstrated no significant difference between groups. In contrast, the affected arm of the VLNT group showed significantly larger circumferences at the level of the wrist (*p* = 0.02) (Table 1, Fig. 1). Similarly, when adjusted for the unaffected arm, the VLNT group also exhibited significantly larger circumferences at the level of the thumb saddle joint (*p* = 0.02), wrist (*p* < 0.001), wrist + 10 cm (*p* = 0.04) and wrist + 20 cm (*p* = 0.02) (Table 2, Fig. 2).

**Table 1:** Mean circumferences of the lymphedematous arms between the VLNT and the LVA groups: *p*-values

Location of measurement	Preoperative	24 Months postoperative
Thumb saddle joint	0.0938	0.988
Wrist	*0.0244*	0.856
Wrist plus 10 cm	0.296	0.518
Wrist plus 20 cm	0.181	0.508
Wrist plus 30 cm	0.591	0.698
Wrist plus 40 cm	0.827	0.988

The values in italic demonstrate statical significance (*p* < 0.05)

**Table 2:** Mean differences of circumferences of the lymphedematous and the unaffected arms between both groups: *p*-values

Location of measurement	Preoperative	24 months postoperative
Thumb saddle joint	*0.0219*	0.501
Wrist	*0.000516*	0.607
Wrist plus 10 cm	*0.0399*	0.444
Wrist plus 20 cm	*0.0186*	0.447
Wrist plus 30 cm	0.111	0.179
Wrist plus 40 cm	0.537	0.251

The values in italic demonstrate statical significance (*p* < 0.05)

**Fig. 3:**
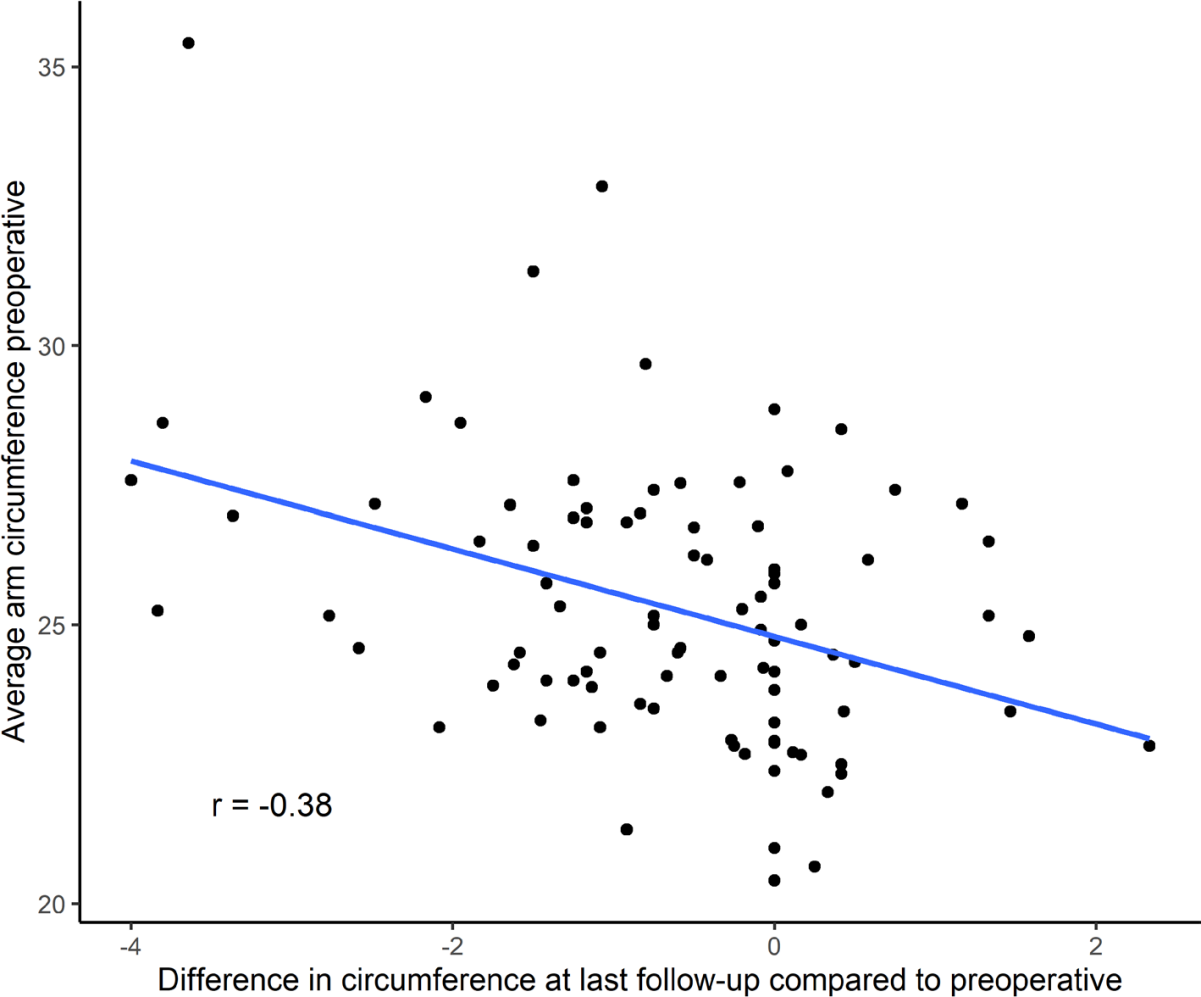
Correlation between preoperative arm circumference and absolute reduction in arm circumference after 24 months

Throughout the 24-month follow-up, both groups displayed significant improvements in arm circumferences. When compared to preoperative values and adjusted for changes in the unaffected arm, VLNT was associated with superior relative reduction rates (RRR) than LVA, revealing RRRs of 2.64 ± 4.64 and 2.14 ± 2.25 (*p* = 0.711), respectively. These findings were confirmed by circumferential measurements, which did not exhibit any remaining significant differences by the end of the follow-up period (Tables 1 and 2). Furthermore, severe cases with larger preoperative circumferences manifested an even more significant improvement of lymphedema (Fig. 3).

Surgery duration was longer in the VLNT group than in the LVA group (199.9 ± 38.6 min versus 169.4 ± 34.5 min, respectively, *p* = 0.08). The occurrence of postoperative complications differed significantly between both groups with a total of 11 (19.0%) complications in the VLNT group, while the LVA group experienced none (Table 3). The majority (63.6%) of complications were minor and classified as Clavien-Dindo II or lower.

**Table 3:** Complications according to Clavien-Dindo in the VLNT and LVA groups

Clavien-dindo	Complication	Therapeutic management		VLNT group	LVA group
		Donor Site Complications	n (%)	6 (10.3)	0
I	Seroma	Needle aspiration	n (%)	5 (8.6)	0
I	Hematoma	Needle aspiration/evacuation	n (%)	1 (1.7)	0
		Recipient Site Complications	n (%)	5 (8.6)	0
IIIb	Fat necrosis	Surgical debridement	n (%)	2 (3.4)	0
IIIb	Partial flap loss	Surgical revision	n (%)	1 (1.7)	0
IIIb	Total flap loss	Surgical revision	n (%)	1 (1.7)	0
II	Erysipela	Antibiotics	n (%)	1 (1.7)	0

## Discussion

### VLNT and LVA: improved patient outcomes

While the circumference of the lymphedematous arm alone provides valuable insights into the severity of chronic BCRL, a comparison of the lymphedematous to the unaffected arm offers a more precise assessment, as it accounts for interindividual factors like BMI shifts over time [25].

Preoperatively, the VLNT group presented with slightly more advanced stages of chronic BCRL than the LVA group, as expressed by larger circumferential measurements of the lymphedematous arm at the wrist (Fig. 1, Table 1). Similarly, the VLNT group also demonstrated significantly larger circumferences at the level of the thumb saddle joint, wrist, wrist + 10 cm and wrist + 20 cm, when adjusted for the unaffected arm (Fig. 2, Table 2). This is expected, as LVA has demonstrated maximum effectiveness in earlier lymphedema stages [34, 35]. It is therefore agreed upon that VLNT is generally reserved for more severe cases of chronic BCRL, which usually present with even larger circumference measurements [35, 36].

Within the two-year follow-up period, both VLNT and LVA significantly reduced arm circumferences. This aligns with the findings of multiple systematic reviews, showing improved subjective and objective patient outcomes after either surgical procedure [15, 20–24]. However, none of these studies directly compared both surgical treatment options. Our novel findings indicate slightly superior relative reduction rates of 2.64 for VLNT, compared to 2.14 achieved with LVA. Moreover, patients with larger preoperative circumferences demonstrated an even greater improvement, suggesting higher effectiveness in more severe cases of lymphedema (Fig. 3).

Lymphedema is traditionally classified into three stages only, ranging from I to III (mild, moderate, and severe, respectively). The preoperative stage of chronic BCRL was documented for 100 out of the 105 patients, with 98 (98%) patients having been categorized as stage II chronic BCRL. The remaining two (2%) patients were classified as stage III chronic BCRL and treated with VLNT. However, it is worth noting that the circumferential measurements indicated a larger preoperative limb volume in the VLNT group, therefore suggesting greater lymphedema severity in this cohort [29, 31, 37].

### Surgery duration and postoperative complications

Accumulating evidence supports correlations between surgical duration and subsequent incidence of postoperative complications and economic costs [38, 39]. In this study, VLNT took 30 minutes longer to perform than LVA. This is likely attributable to the two-team approach, where surgeries were performed concurrently with one team harvesting nodes, and another team implanting nodes at a high-volume lymphatic center. Nevertheless, the longer duration of VLNT does appear to correlate with an increased complication rate: while there were 11 (19.0%) occurrences in the VLNT group, the LVA experienced none. Six (10.4%) of these complications arose at the donor site and 5 (8.6%) at the recipient site, which mirrors complication rates in current literature [21, 22, 24]. Most complications were grade II or lower according to Clavien-Dindo (Table 3) [32]. As such, the data of this study suggests a higher occurrence of postoperative complications in the VLNT group, which is conceivable, given that VLNT necessitates two surgical sites as opposed to one in LVA.

### Study strengths and limitation

This study’s main strength is its large sample size of 105 patients. This is noteworthy for a study in the field of lymphatic surgery and largely unrivaled by other studies on VLNT and LVA, as demonstrated in various systematic reviews [15, 18, 40].

The main limitation of this study is its retrospective design. Furthermore, future research should incorporate validated patient-reported outcome measures (PROMs), such as the LYMPH-ICF or LYMPH-QOL, alongside traditional clinical parameters like circumference measurements [41–43]. Given that the primary objective of lymphatic surgery is improving patient quality of life, systematically incorporating PROMs in future research is essential. Despite the increasing integration of PROMs into clinical practice, the implementation has only recently begun to gain momentum, therefore excluding their application in this retrospective analysis. Circumference measurements are a widely used informative and cost-effective option with proven reliability [44–46]. Nonetheless, they fail to correlate with quality of life (QoL)-related outcomes and thus do not adequately portray the burden of chronic BCRL from the patient’s perspective [43, 47]. Therefore, prospective trials integrating validated PROMs in the pre- and postsurgical setting may deepen our understanding on the impact of supermicrosurgical procedures in the treatment of chronic BCRL [48].

## Conclusions

This study reveals that both VLNT and LVA are safe procedures that lead to improved outcomes in patients with chronic BCRL. However, VLNT demonstrated an increased relative reduction rate in more advanced stages of BCRL at the price of an increased risk of (predominantly minor) postoperative complications. We recommend PROMs to be implemented into routine clinical practice to evaluate the patient’s perspective as the most important clinical outcome measure after lymphatic surgery.

## Data Availability

The data within our encrypted databank that was analyzed as part of this study is not publicly available pursuant to the ethics committee approval by the “Ethikkommission Nordwest und Zentralschweiz EKNZ” in Switzerland due to their sensitive nature. Nevertheless, the data can be obtained from the corresponding author on reasonable request.
